# Gut, oral and skin microbiome of Indian patrilineal families reveal perceptible association with age

**DOI:** 10.1038/s41598-020-62195-5

**Published:** 2020-03-30

**Authors:** Diptaraj S. Chaudhari, Dhiraj P. Dhotre, Dhiraj M. Agarwal, Akshay H. Gaike, Devika Bhalerao, Parmeshwar Jadhav, Dattatray Mongad, Himangi Lubree, Vilas P. Sinkar, Ulhas K. Patil, Sundeep Salvi, Ashish Bavdekar, Sanjay K. Juvekar, Yogesh S. Shouche

**Affiliations:** 1 National Centre for Microbial Resource, National Centre for Cell Science, Central Tower, Sai Trinity Building Garware Circle, Sutarwadi, Pashan, Pune, India; 2 Vadu Rural Health Program, KEM Hospital Research Centre, Pune, India; 3 R. C. Patel ASC College, Shirpur, Dhule India; 4 Chest Research Foundation, Marigold Premises, Survey no 15, Kalyaninagar, Pune, India; 5 KEM Hospital, Pediatrics Department, KEM Hospital, Rasta Peth, Pune, India; 6 Department of Microbiology, Government Institute of Science, Aurangabad, India

**Keywords:** Microbial ecology, Molecular ecology

## Abstract

The human microbiome plays a key role in maintaining host homeostasis and is influenced by age, geography, diet, and other factors. Traditionally, India has an established convention of extended family arrangements wherein three or more generations, bound by genetic relatedness, stay in the same household. In the present study, we have utilized this unique family arrangement to understand the association of age with the microbiome. We characterized stool, oral and skin microbiome of 54 healthy individuals from six joint families by 16S rRNA gene-based metagenomics. In total, 69 (1.03%), 293 (2.68%) and 190 (8.66%) differentially abundant OTUs were detected across three generations in the gut, skin and oral microbiome, respectively. Age-associated changes in the gut and oral microbiome of patrilineal families showed positive correlations in the abundance of phyla Proteobacteria and Fusobacteria, respectively. Genera *Treponema* and *Fusobacterium* showed a positive correlation with age while *Granulicatella* and *Streptococcus* showed a negative correlation with age in the oral microbiome. Members of genus *Prevotella* illustrated high abundance and prevalence as a core OTUs in the gut and oral microbiome. In conclusion, this study highlights that precise and perceptible association of age with microbiome can be drawn when other causal factors are kept constant.

## Introduction

Aging is an extremely complex, perpetual, progressive and multifactorial process resulting in decreased physiologic functions of all the organ systems^1^. Studies have reported that the human microbiome, the latest discovered organ, is also significantly influenced by increasing age^2–4^. Studies have suggested that the complex and diverse communities of microbes that inhabit the gut vary through different stages of an individual’s life^5^. Many of these alterations are harmless and natural, while some of the alterations can have an important effect on overall homeostasis^6^. Importantly, notable changes in the human microbiome occur when the immune system is relatively weak, i.e., at the start of life and during aged life^7^. Many of the fluctuations in the microbiome are harmless and natural; nonetheless, studies have shown that some disturbances in the gut microbiome can have important effects on health and disease^6^. Earlier studies on human microbiome analyses have discovered an increased abundance of *Bacteroides* species in the elderly population^8^. An enriched abundance of Proteobacteria a bacterial group containing ‘pathobionts’ known for causing impairment in a susceptible host, i.e., in centenarian’s^2–4^. But the populations used for these studies were either from different geographical locations or with different diets^2–4^. In agreement with the studies on the human gut microbiome, in older micro-pigs^9^ also decreased abundance of beneficial microbes including probiotic bacteria and short-chain fatty acid-producers was recorded while *Bacteroides* were increased with the increasing age^9^.

Several studies have shown age-associated effects in the experimental animals wherein, transfer of an aged microbiome into germ-free mice leads to systemic inflammation^10,11^, on the contrary replacement of microbiome of the aged mice with the microbiome of younger mice boosts the local germinal center reactions^1^. Researchers noted that the aged mice’s germinal center reaction was stronger following fecal transplantation from the younger mice^1^. Moreover, remodeling of the gut microbiome has shown to increase the lifespan in diverse organisms, including *Drosophila melanogaster*^12,13^, killifish^14^, mice^15^, etc.

Coevolved with the host and its ancestors for millions of years, the microbiome plays a key role in the maintenance of host health and wellbeing by performing various functions ranging from digestion, protection against pathogen colonization to host immunity and regulation of central nervous^16–21^. Also, the microbiome is responsive to a variety of confounding factors such as host ethnicity^22,23^, age^24,25^, diet^26,27^ and geographic location^24,28^. Hence, it is imperative to study the same population longitudinally for the exploration of a precise association of age and microbiome. Studying the microbiome of genetically linked individuals of multiple generation families having similar diet, ethnicity and staying in the same geographic regions can help in estimating the perseverance of microbiota obtained in early years and its progression with age. Traditionally, India has an established convention of the joint family system, which is an extended family arrangement consisting of three or more generations living in the same household structure bound by genetic relatedness^29,30^. Especially in rural and semi-urban joint families in India, family members live in the same house structure and have similar dietary and sanitary habits. Hence, the Indian population provides a unique opportunity to understand age-related changes in the human microbiome. Studies relating to the influence of FUT2 and birth mode variants on microbiome^31^, diabetes-associated microbiome^32^, obesity-related microbiome^33^, the microbiome of celiac disease patients^34^, an association of microbiome with ayurvedic Prakriti types^35^, microbiome structure of rural, urban^36^ and tribal^37^ populations were carried out in the Indian populations. However, age-related changes in the human microbiome across different body habitats are unknown. The majority of studies understanding the age and human microbiome correlations have been focused on the gut microbiome, while changes in oral and skin microbiome in the aging process have been relatively less studied.

In the present study, we provide a comprehensive analysis of the human microbiome from the gut, oral and skin ecosystems from 54 healthy subjects belonging to six different patrilineally related three generation families staying together in rural Indian settings. The study population has harmonized dietary, social habits, hygiene and sanitation habits, economic status and geographic position. Predominantly, other microbiome contributing factors were harmonized and age was the only distinguishing factor. 16S rRNA gene amplicon sequencing-based microbiome analysis performed to investigate the age-related changes in the gut, skin and oral microbiome of Endogamous Agriculturist Indian (EAI) sub-population.

## Results

### Gut, oral and skin microbiome profile of EAI population

Overall, a total of 9,566,497 sequences were generated, out of which 8,048,975 (File S1) were taxonomically assigned, resulting in 6,708 OTUs for the gut microbiome. Four samples (St.D1004, St.D301, St.S610 and St.S612) were removed from further analysis due to lower sequencing depth. Bacterial phyla such as Bacteroidetes (49.3%), Firmicutes (41.6%), Proteobacteria (5.7%), Actinobacteria (2.18%) and Tenericutes (0.4%) were highly dominant and constituted ~99% of the total gut microbiome. Presence of 174 different bacterial genera was noted, wherein *Prevotella* (50%), *Dialister* (12%), *Bacteroides* (9%), *Megamonas* (3%), and *Succinivibrio* (3%) were among the dominant taxa contributing to a total of 77% of the gut microbiome (Fig. S1a). Although, genus *Prevotella* was observed to have varying relative abundance ranging from 2% to 77% across the study population, its dominance was evident from the fact that 62% (n = 31) of the study subjects had an abundance in a range of 33% to 77% of the total gut microbiome (Table S1).

In the oral microbiome, 7,568,649 good quality sequences (File S1) clustered into 2,167 OTUs. Analysis based on the taxonomic assignment of these reads revealed a higher abundance of bacterial phyla such as Proteobacteria (34%), Bacteroidetes (32%), Firmicutes (24%), Fusobacteria (6%) and Actinobacteria (2%) constituting 96% of the oral microbiome. Genera *Neisseria* (20%), *Streptococcus* (15%), *Prevotella* (14%), *Porphyromonas* (10%), and *Haemophilus* (10%) were found to be the five most dominant genera totaling up to 69% of the oral microbiome (Fig. S1b). The relative abundance of genus *Prevotella* was observed to be more than 10% in half of the population (Table S2).

**Figure 1:**
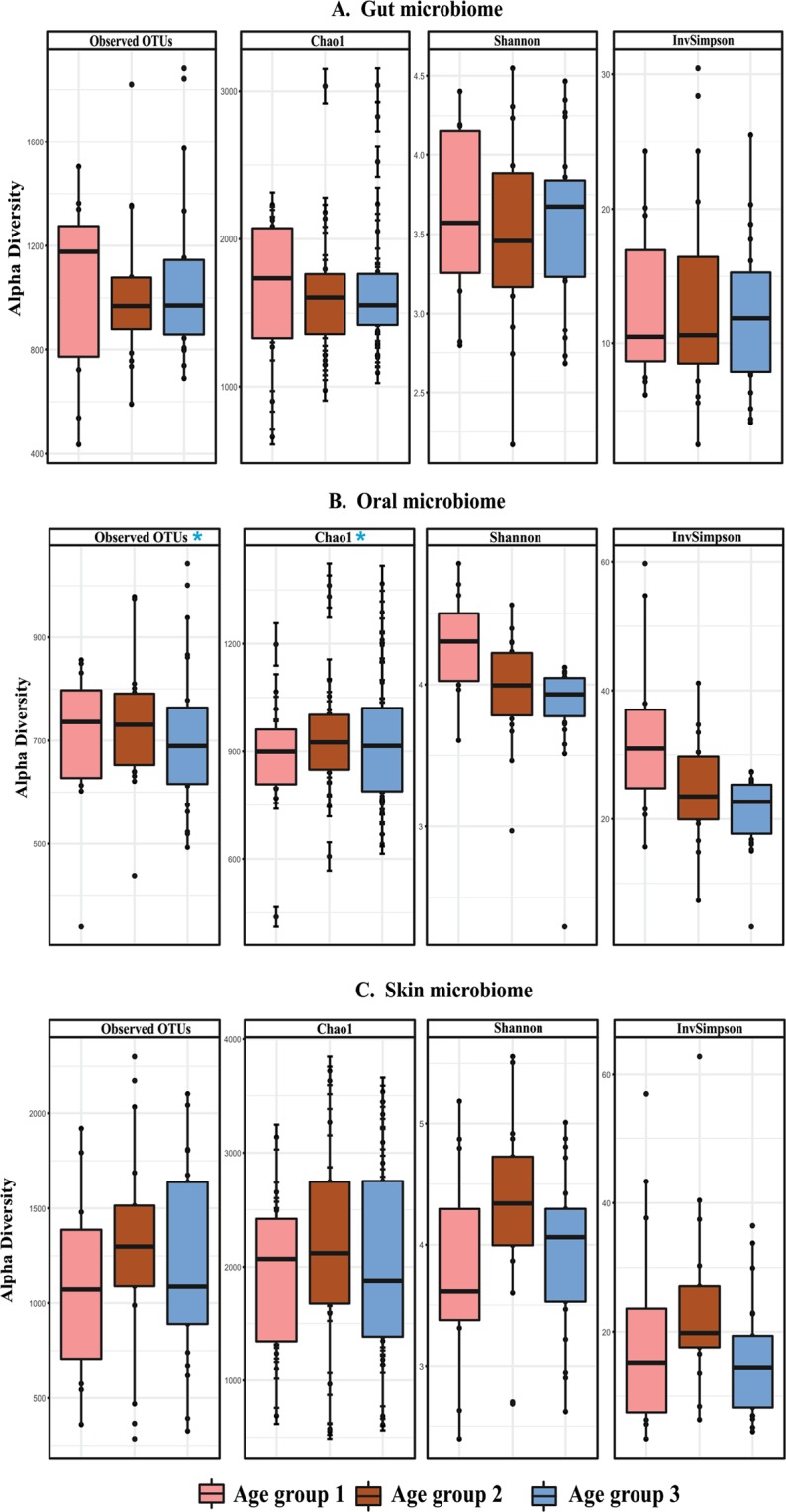
Boxplot of alpha diversity measures across the three generations (age groups) in the gut (**A**) oral (**B**) and (**C**) skin samples. The boxes denote interquartile ranges (IQR) with the median as a black line.

The skin microbiome data comprised of 10,951,175 good quality sequences (File S1) clustered into 10,920 OTUs. Skin microbiome analysis showed a higher abundance of phyla Firmicutes (49%), Proteobacteria (26%), Actinobacteria (12%) and Bacteroidetes (8%) collectively on 11 different body sites including dry, moist and sebaceous regions. Only one percent of OTUs were assigned to phylum Cyanobacteria. The skin microbiome showed the highest number of OTUs (n = 10,920) compared to oral and stool samples. *Corynebacterium* (10%) *Alloiococcus* (9%), *Staphylococcus* (8%), *Streptococcus* (7%) and *Anaerococcus* (6%) were the most dominant and diverse bacterial genera detected in the skin microbiome (Fig. S1c). Alpha diversity analysis measures, i.e., observed species (OTUs), Chao1, Shannon and Inverse Simpson revealed no significant differences in the gut and skin microbiome when compared between the three age groups (Fig. 1), family structure and dietary habits (Table 1). However, significant differences were observed in the oral microbiome between the age groups (ANOVA, p < 0.05 with Benjamini-Hochberg FDR corrections) (see Fig. 1B).

**Table 1:** The table illustrates the diversity indices calculated for the gut, oral and skin samples.

Sample Type	Description	Samples Nos.	Chao1 (Average)	Goods coverage (Average)	Observed species (Average)	PD whole tree (Average)	Shannon (Average)	Simpson (Average)
Human gut	Age group 1	11	2020.02 ± 466.57	0.996 ± 0.996	1314.73 ± 1314.73	64.92 ± 64.92	5.29 ± 5.29	0.91 ± 0.91
	Age group 2	18	1907.05 ± 635.75	0.996 ± 0.003	1228.89 ± 426.12	61.11 ± 17.02	4.88 ± 1.05	0.86 ± 0.13
	Age group 3	23	2123.67 ± 539.53	0.996 ± 0.001	1378 ± 368.18	66.93 ± 15.07	5.17 ± 0.74	0.9 ± 0.9
Human skin	Age group 1	12	2883.26 ± 1038.83	0.99 ± 0.007	1853.25 ± 831.07	107.51 ± 35.29	5.47 ± 1.3	0.91 ± 0.07
	Age group 2	17	3293.69 ± 1147.45	0.992 ± 0.007	2310.47 ± 880.29	122.83 ± 34.75	6.2 ± 1.15	0.95 ± 0.03
	Age group 3	23	3054.99 ± 1398.22	0.977 ± 0.078	2135.13 ± 1098.27	113.52 ± 46.59	5.7 ± 0.97	0.91 ± 0.05
Human oral	Age group 1	12	**736.55** ± 141.25*	0.999 ± 0.001	**607.67** ± 116.94*	32.33 ± 4.74	6.11 ± 0.57	0.96 ± 0.02
	Age group 2	18	**800.83** ± 160.61*	0.999 ± 0	**629.61** ± 109.04*	33.84 ± 7.94	5.81 ± 0.47	0.96 ± 0.02
	Age group 3	24	**809.69** ± 200.33*	0.999 ± 0	**611.5** ± 140.96*	33.52 ± 9.85	5.64 ± 0.54	0.95 ± 0.06

*Statistically significant differences across the generations.

### Contribution of core taxa in the gut, oral and skin microbiome of patrilineal families

**Figure 2:**
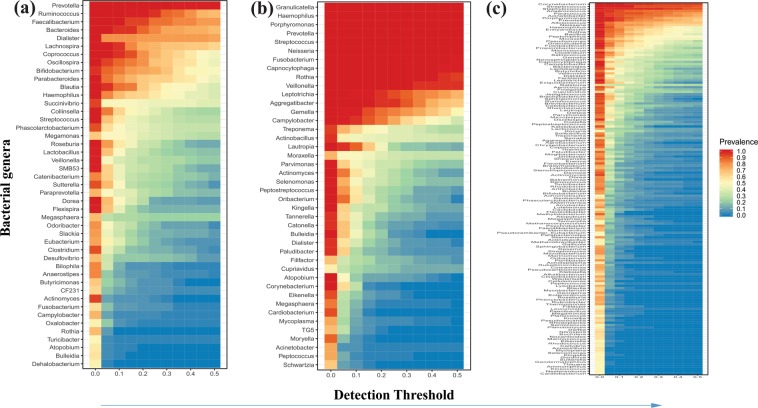
Heatmap representing the core bacterial genera detected across the gut (**a**), oral (**b**) and skin (**c**) microbiome samples of the EAI population.

Bacterial genera prevalent in 95% of the study population with more than 0.1% abundance were considered as a part of the core microbiome. Estimation of core microbiome was performed for individual families (n = 6 families) and the overall EAI study population. Amongst the 171 total bacterial genera detected in the gut microbiome, only three genera, namely *Prevotella, Ruminococcus* and *Faecalibacterium*, were recorded as a part of core microbiome across all the families (Fig. 2a, Table S3). These core taxa represented 23% to 91% gut microbiome composition of the participants (Fig. S2). With the aforementioned detection threshold few bacterial genera were explicitly detected in particular family as core taxa wherein, *Parabacteroides* was detected in family D3, *Haemophilus* and *Roseburia* in family D8, *Streptococcus* and *Dorea* in family D10 (Fig. S3, Table S3).

The core microbiome of oral samples represented presence of 13 (6.7%) bacterial genera amongst the 192 total genera. These genera include *Neisseria, Streptococcus, Prevotella, Porphyromonas, Haemophilus, Fusobacterium, Granulicatella, Veillonella, Capnocytophaga, Rothia, Aggregatibacter, Gemella* and *Lautropia* (Fig. 2b). Overall, these core taxa estimated 79% to 96% of the total microbiome composition (Fig. S2).

In the skin microbiome samples, *Corynebacterium* and *Streptococcus* were the only bacterial genera detected as core taxa across all the families (Fig. 2c, Table S5). Similar to the gut microbiome, family-specific bacterial genera were also detected in the skin microbiome. These genera include *Novosphingobium* in family D8, *Enhydrobacter, Salinicoccus* and *Butyrivibrio* in family D10, and *Haemophilus* and *Gemella* in family S6 (Fig. S5, Table S5). These core taxa represented 37% to 94% of the total microbiome (Fig. S2). Largely, the contribution of core taxa in the gut, oral and skin microbiome of all the patrilineal families was identical.

### Influence of diet on the gut microbiome

**Figure 3:**
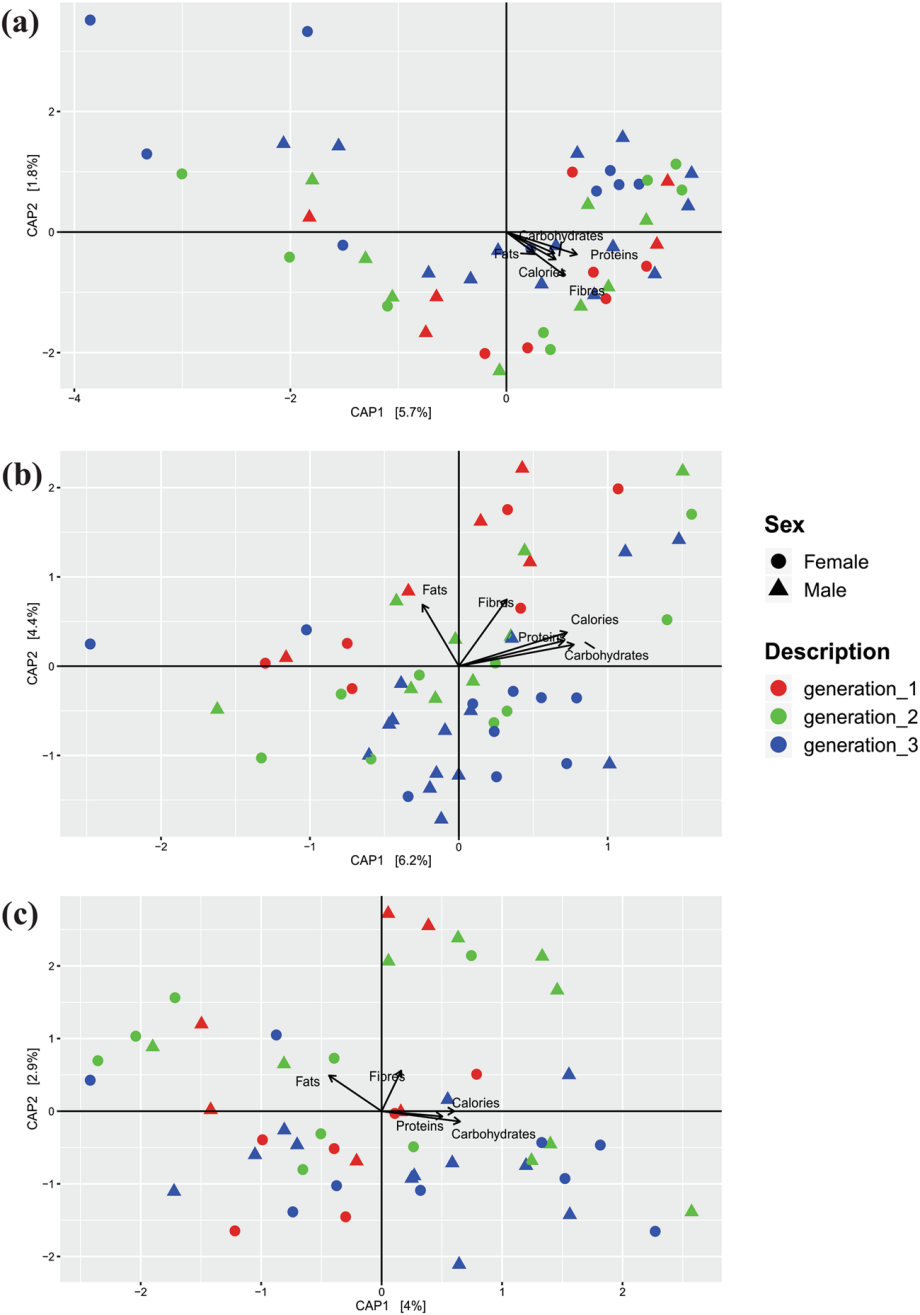
Canonical correspondence analysis (CCA) plot of bacterial genera and age group relationship calculated for gut (**a**) oral (**b**) and skin (**c**) microbiome of the endogamous agriculturist Indian subpopulation.

**Figure 4:**
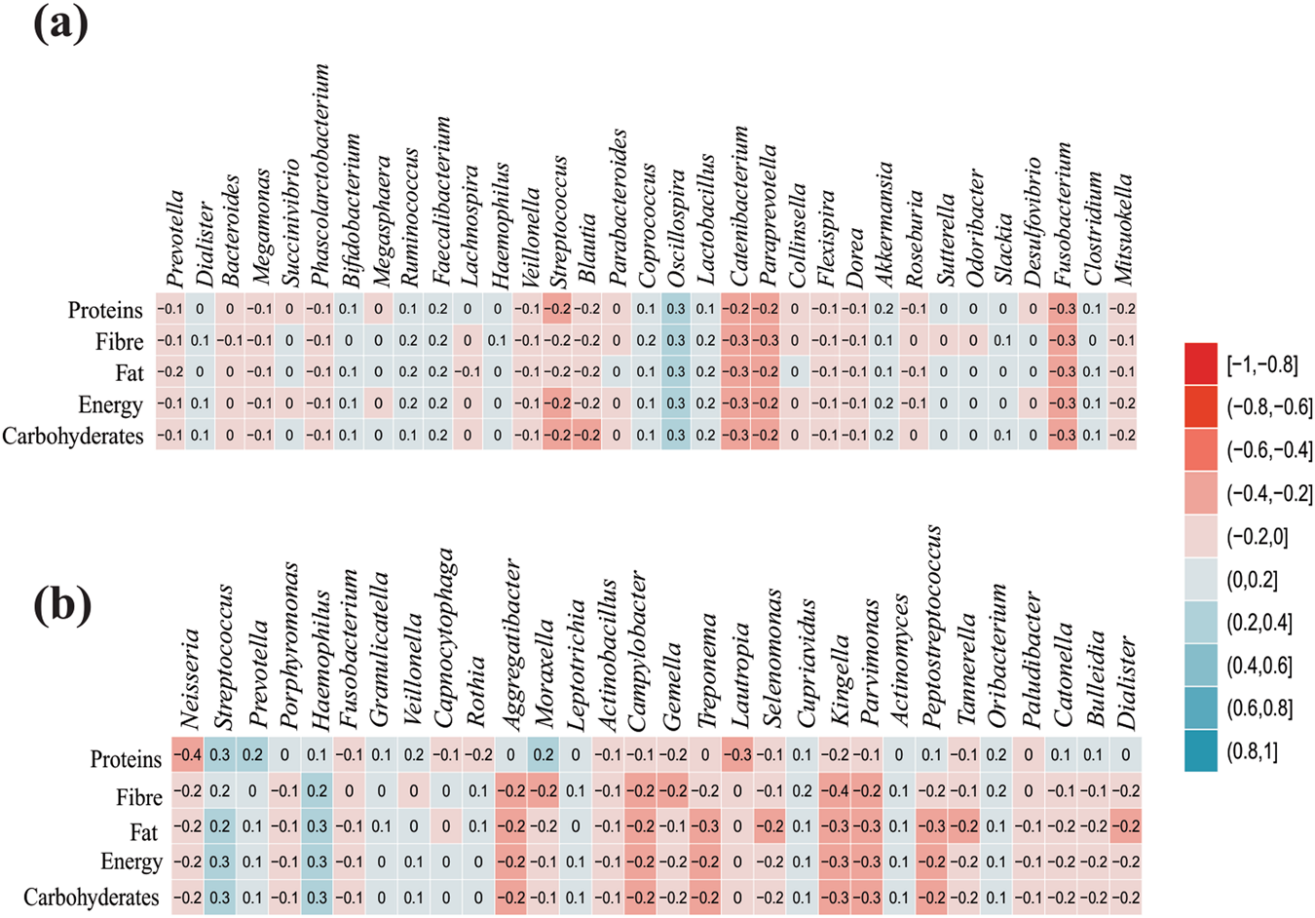
Correlation analysis of microbiome (genus levels) and dietary consumption of carbohydrates, proteins, fats, fibers and calories for the human gut (**a**) and oral (**b**) microbiome.

Detailed dietary information of the study population was collected using the food frequency questionnaire (FFQ). With the help of a nutritionist, the dietary information was subsequently converted into the daily intake of carbohydrates, proteins, fats, lipids, fibers and calories (Table S6). Investigation revealed that average carbohydrates consumption in the first, second and third-generation members was 166, 396 and 339 grams, providing 74%, 81% and 80% of daily calories in the respective generations. Overall the type and amount of dietary components were similar across the population, except for family D3, which has relatively lesser consumption of these components. Canonical correspondence analysis (CCA) based on the abundance of bacterial genera, amount of dietary components and samples metadata showed that all the samples were scattered across the ordination plot and no clear clustering of the samples was observed based on age group or gender (Fig. 3). The variation explained by the ordination plot was also non-significant, reporting 7.5% for the gut, 10.6% for the oral and 6.9% for the skin microbiome (Fig. 3). Also, correlation analysis between the relative abundance of bacterial taxa and routine consumption of dietary components showed no significant association (Fig. 4).

### Association of age and microbiome

**Figure 5:**
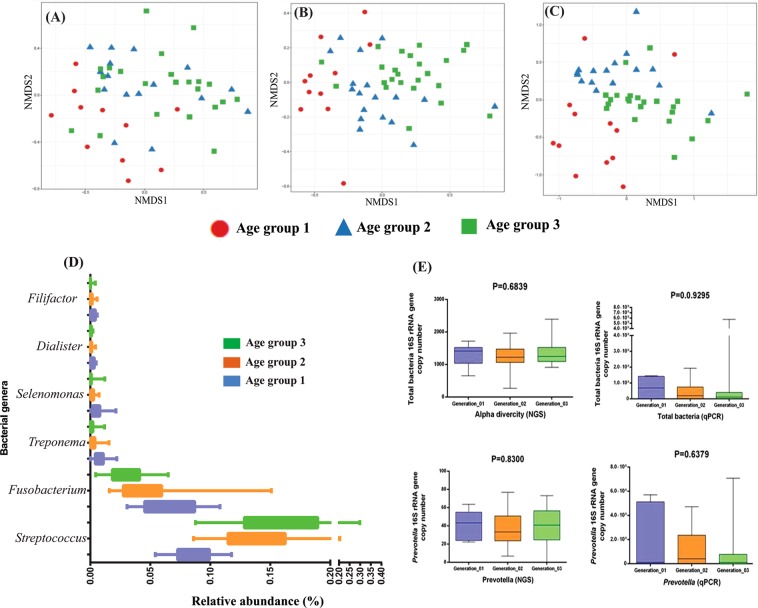
Nonmetric Multidimensional Scaling (NMDS) ordination displaying microbiome communities across the three generations in the gut (**A**), oral (**B**) and skin (**C**) microbiome. (**D**) Box plot showing differentially abundant genera in the human oral microbiome across the members from the three age groups. (**E**) Next-generation sequencing and qPCR results showing the abundance of *Prevotella* and total bacteria in the human gut microbiome across three age groups.

Microbiome community structure of gut, oral and skin samples was investigated across three generations (age groups). Amongst the prevalent bacterial genera of the gut microbiome, *Succinivibrio* and *Ruminococcus* were highly abundant in the age group 1, *Dialister, Megamonas, Phascolarctobacterium, Megasphaera* and *Faecalibacterium* in the age group 2 and *Prevotella, Bacteroides* and *Bifidobacterium* were in the age group 3 (Table S7a). Likewise, in the oral microbiome *Prevotella, Fusobacterium, Veillonella, Capnocytophaga, Rothia* and *Aggregatibacter* were highly abundant in the age group 1, genus *Haemophilus* in age group 2 while *Neisseria, Streptococcus, Porphyromonas* and *Granulicatella* in the age group 3 (Table S7b). High abundance of few bacterial taxa was recorded in particular age groups in the skin microbiome samples also, wherein *Corynebacterium, Alloiococcus, Peptoniphilus, Haemophilus, Acinetobacter* and *Clostridium* were highly abundant in age group 1, *Anaerococcus, Porphyromonas* and *Campylobacter* in age group 2 and *Staphylococcus, Streptococcus, Novosphingobium, Paracoccus, Moraxella* and *Prevotella* in age group 3 (Table S7c). Primarily, age-associated changes were observed in the microbiome structure of three-generation members and to strengthen these observations; statistical analysis was also completed. Comparative microbiome analysis in three age groups showed no significant differential abundance of bacterial genera in the gut and skin microbiome. However, the oral microbiome showed significant variations in the abundance of genera *Dialister*, *Fusobacterium, Streptococcus, Selenomonas, Filifactor* and *Treponema* (Fig. 5D) (ANOVA, p < 0.05 with Benjamini-Hochberg FDR corrections). We confirmed our observations using qPCR analysis for quantifying the absolute proportion of genus *Prevotella* in the total human gut bacteria (Fig. 5E).

Beta diversity analysis using non-metric multidimensional scaling (NMDS) plots based on Bray-Curtis metrics showed no clear clustering in the samples based on the age groups of the study population (Fig. S6). Age-associated changes in the microbiome were further analyzed based on differentially abundant OTUs (ANOVA, p < 0.05 with Benjamini-Hochberg FDR corrections). Investigation revealed the presence of 69 (1.03%) differentially abundant OTUs across three age groups in the gut microbiome. Similarly, 190 (8.66%) and 293 (2.68%) differentially abundant OTUs were observed in human oral and skin microbiome, respectively. A high number of differentially abundant OTUs were present in the oral samples. Beta diversity analysis using these differentially abundant OTUs showed clustering of samples based on the age groups in the gut, oral and skin samples (Fig. 5A–C).

**Figure 6:**
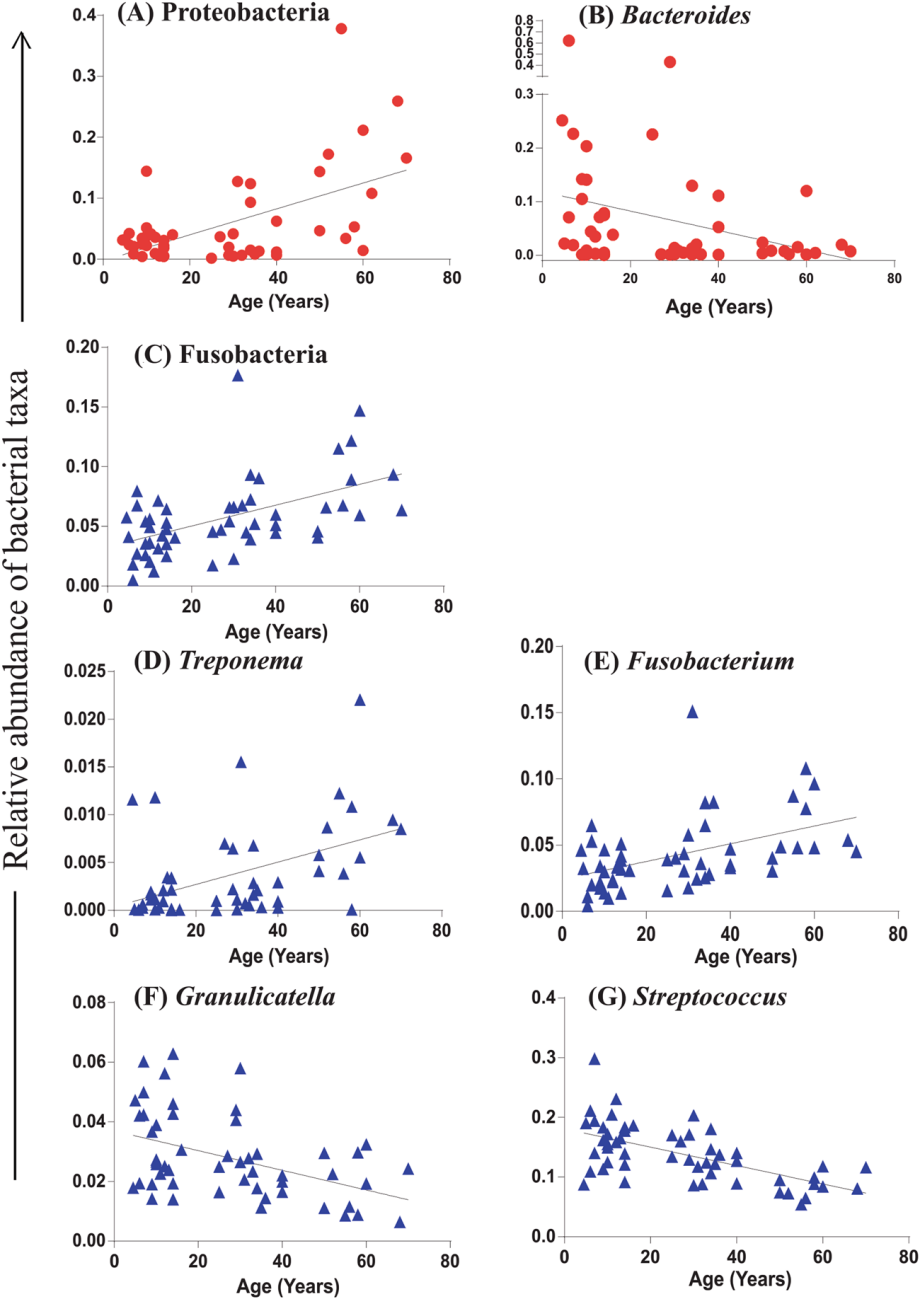
Correlation analysis of bacterial abundance with age, Phylum *Proteobacteria* (**A**) and Genus *Bacteroides* (**B**) of gut microbiome; Phylum *Fusobacteria* (**C**), genera *Treponema* (**D**), *Fusobacterium* (**E**), *Granulicatella* (**F**) and *Streptococcus* (**G**) of oral microbiome (p =< 0.05, r^2^ > 0.2 for all the correlation).

We further performed a correlation analysis of gut, oral and skin microbiome with age. Linear regression analysis using the nonparametric Spearman correlation revealed a higher abundance of phylum *Proteobacteria* with increasing age in the gut microbiome (p < 0.05) (Fig. 6A). While, in the oral microbiome of the population, a higher abundance of phylum *Fusobacteria* was observed with the increasing age (p < 0.05) (Fig. 6B). However, no such age-based correlations were observed in the skin microbiome.

Amidst the total 171 bacterial genera in the gut microbiome, only genus *Bacteroides* showed age-associated changes. Decreased abundance *Bacteroides* was recorded with the increasing age (nonparametric Spearman correlation (p < 0.05) (Fig. 6B). Whereas, in the oral microbiome of the population, bacterial genera *Treponema* and *Fusobacterium* showed a positive correlation (Fig. 6D,E) while genera *Granulicatella* and *Streptococcus* showed a negative correlation with the age (p < 0.05) (Fig. 6F,G). However, in the skin microbiome, no such statistically significant correlations were noted.

## Discussion

Indian patrilineal extended family structure provides a unique opportunity to study the underlying effects of age, diet, and genetics influencing the human microbiome. Such family structure is a widely seen residential unit comprising of 2–4 patrilineally related generations living together, particularly in rural and semi-urban settings^29,30^. In the present study, we analyzed gut, oral and skin microbiome from 54 healthy subjects belonging to six different families from a single biogeographic region (Dongargaon: 18.6199° N, 74.0807° E and Shikrapur: 18.6924° N, 74.1323° E).

In the gut and oral microbiome, we found a high prevalence of *Prevotella* (Fig. 2a,b; Tables S1 and S2), a bacterial genus known to be associated with degradation of complex plant polysaccharides^38,39^. Indian diet is rich in plant-based carbohydrates, and our observations are consistent with earlier reports where a high prevalence of *Prevotella* in the gut microbiome of the Indian population was observed^39,40^. Prevalence of *Prevotella* has also been reported in the African population consuming a diet rich in carbohydrates and fibers^41^.

Understanding the confounding factors that shape and define the oral microbiome is crucial for understanding the broader systemic health^42^, as oral microbiome has long been known to be a reservoir for infection at other body sites^43^. In our analysis, high abundance of bacterial genera *Neisseria*, *Streptococcus*, and *Prevotella* were observed (Fig. S1b). Genus *Neisseria* is aerobic and primary colonizers of the oral cavity, *Streptococcus* is a facultative anaerobe while *Prevotella* is the obligate anaerobic bacteria. The high abundance of aerobic, facultative anaerobic and obligate anaerobic bacteria suggests the role of oxygen sensitivity in structuring the composition of bacterial diversity associated with the oral cavity. This diverse microbiome can perform versatile metabolic functions crucial for the healthy oral cavity. Earlier, these bacterial genera reported being common residents of the oral cavity in different populations in the healthy state^44–47^.

Investigating influencing factors is important to determine homeostatic forces that contribute to a healthy skin microbial community^48^. In the skin microbiome, *Corynebacterium*, *Alloiococcus*, and *Staphylococcus* were found to be the most abundant genera (Fig. S1c). These observations are in agreement with those reported by earlier studies in diverse ethnic groups, globally^49^. In contrast with an earlier study that reported Actinobacteria as the most dominant phylum of the skin microbiome^50^, we observed dominance of Firmicutes followed by Proteobacteria and then Actinobacteria. These differences in skin microbiome of EAI sub-population could be associated with unique genetics, ethnicity and environmental conditions.

Further, different regions of the human skin like dry, moist and sebaceous are known to harbor different microbial community^51^. These differences could not be ascertained in our study since skin samples collected from 11 different body sites were pooled together before sequencing. The skin of healthy individuals generally harbors low microbial biomass and it requires sufficient starting material^52^. Hence DNA from all the 11 body locations of the participants were extracted separately and eventually pooled together before sequencing to avoid sequence artifacts associated with low biomass samples^52^.

Microbial diversity, which contributes to the core microbiome, can provide a snapshot of homeostasis in the population and deviations from this core can be associated with different physiological states^53^. Presence of three, 13 and two core genera were observed in the gut, oral, and skin microbiome, respectively (Fig. 2). Genus *Dialister* was also amongst the highly abundant core taxa of the gut microbiome in all the families of the EAI population (Figs. 2, S3). An earlier study has reported that microbial enzymatic repertoire is known for the conversion of dietary fibers into short-chain fatty acids (SCFA)^54^. Our observations of a higher abundance of *Dialister* in Indian sub-population along with high consumption of dietary fiber suggests a need to test this possible association, as these bacteria are previously reported for SCFA production (propionate)^55^. Gut and oral microbiome showed a high proportion of core microbiome compared to the skin microbiome. Amongst the core taxa in oral microbiome, *Porphyromonas* are known for the expression of the *fimA* gene, which encodes for the surface protein important for attachment to other oral bacteria^56^. The oral microbial flora comprises diverse human-associated biofilms, which are influenced by oral *streptococci*, the main group of early colonizers^57^. *Fusobacterium* was also prevalent taxa in the oral microbiome. In the complex ecosystems like oral cavity microbial co-aggregations like *Fusobacterium* and *Streptococcus*, which mediates a variety of cooperative metabolic functions^57^. Skin is the largest organ and represents the primary physical barrier between the host and the external environment. The overall representation of the core microbiome was only 15%, presumably due to oil, moist and sebaceous site-specific bacterial community structure and transient nature of the skin microbiome. Due to the acidic pH of the skin (4.4 to 5), despite the transient nature of skin microbiome, only mutualistic skin bacteria like *Streptococcus*, *Staphylococcus* and *Corynebacterium* can grow and detected as core taxa. Unique combination of taxa such as *Dialister, Prevotella, Bacteroides, Megamonas, Succinivibrio* in gut, *Streptococcus, Fusobateria Neisseria, Prevotella, Porphyromonas* in oral and *Corynebacterium, Alloiococcus, Staphylococcus, Streptococcus, Anaerococcus* and *Peptoniphilus* in skin were observed in EAI subpopulation emphasizing the effect of diet, host genetics and environmental factors on microbiome. Overall the core microbiome structure of the EAI population was similar across all the families which can be associated with similar dietary patterns, socioeconomic status, ethnicity, and agriculture-based lifestyle. Few low abundant bacterial taxa were exclusively detected as core taxa of specific families (Tables S3–S5). However, no relatable information was observed for the distinctive presence of these taxa.

Dietary information of the study population was collected using the food frequency questionnaire (FFQ) and this information is subsequently translated into the daily intake of carbohydrates, proteins, fats, lipids, fibers and calories with the help of a nutritionist. Detailed analysis showed that carbohydrates provide about 74%, 81% and 80% calories in the first, second and third generation members, respectively (Table S6) and overall, the consumption of other dietary constituents was comparable across the three generations. Correlation analysis of bacterial genera and routine dietary consumption of carbohydrates, proteins, fats, lipids, fibers and calories showed that there is no statistically significant correlation suggesting the relatively similar structure of microbiome and overall dietary pattern (Fig. 4). This observation is further strengthened by limited variation observed in the CCA analysis and no specific clustering was observed based on the generation (age groups) or the gender of the study participants (Fig. 3). A balanced diet helps in maintaining human health and the changes in the diet are responsible for the associated alterations in the microbiome. Singh *et al*., have shown that dietary alterations can induce microbiome associated changes in 24 hours, which can be alternating and yet reproducible^58^. Patrilineal families in this study follow the typical diet for several generations, and generally, all members of the family eat the same food irrespective of their age. The routine diet of the study populations comprises majorly of wheat and/or pearl millet bread, rice, vegetables and millets. The correlation analysis on this population revealed no statistically significant differences in the microbiome and the diet. This emphasizes the fact that overall homogeneity in the diet helps in maintaining the microbial state. Other confounding factors, including birth mode (cesarean section delivery and normal delivery), monozygotic or dizygotic twins had no effect on the microbiome as all the participants recruited in the study have the same normal delivery birth mode and none of the participants were twins.

A substantial number of studies have reported the association between age and the human microbiome^59,60^, but the majority of these studies were among unrelated individuals who lacked constant causal contributing factors. Participants from three generations belonging to patrilineal families and living in the same household were recruited in this study to understand the perceptible effect of age on the microbiome. This sampling strategy allowed us to have a minimum impact of other confounding factors on the microbiome. Human microbiome dynamics changes with the time as the ‘holobiont’ integrates and responds to signals from the environment^61^. A direct causal relation between age-specific microbial communities and host aging has also been explored in laboratory model organisms, including flies, fish and mice^1,12–16^, etc. Microbiome community structure of gut, oral and skin samples illustrated differences in the abundance of bacterial genera in three age groups. Here, *Succinivibrio* known for higher fiber degrading potential^62^ and *Ruminococcus* were highly abundant in first-generation members (Table S7a). The specific reason for the higher abundance of these taxa is not known and it demands further investigation. Bacterial taxa known for healthier metabolism were abundant in the gut microbiome of the second generation members such as *Dialister* and *Phascolarctobacterium* the SCFA producers^63,64^, *Megasphaera* the key carbohydrate metabolizing bacteria of Indian population known for having diverse and unique sets of Carbohydrate-Active enzymes (CAZymes)^65^, *Faecalibacterium* is also the most abundant and important commensal bacteria of the human gut microbiota^8^. In addition to *Prevotella, Bifidobacterium*, the early gut colonizers and *Bacteroides* were higher in the third generation members (Table S7a). In the skin microbiome also age-related changes in the abundance of bacterial taxa were recorded. Genus *Corynebacterium* was highly abundant in first-generation members (Table S7c). Recent study understanding the extrinsic and intrinsic host factors influencing skin microbiome composition suggested that *Corynebacterium* OTUs were associated with skin aging^66^, specifically with the hyperpigmented spots and wrinkles^66^. With the increasing age, physiological changes occur in the skin structure explains the association of key bacterial taxa in the members of the respective age groups. In our study, only 1.03% OTUs were found to be differentially abundant across three age groups, suggesting a nominal but profound effect of age on the gut microbiome. With the increasing age, the high abundance of Proteobacteria was detected (Fig. 6A). A higher abundance of this bacterial phylum was reported to be associated with the altered gut microbiome and dysbiosis^67,68^. Studies have shown an increase in the abundance of Proteobacteria with age correlating with the weaker immune response to the opportunistic pathogens, thereby leading to a decrease in the commensal microflora^69,70^. Proteobacteria have been suggested as the potential diagnostic criteria for dysbiotic conditions^7^.

Similarly, in the oral microbiome, *Fusobacteria* was found to increase with increasing age (Fig. 6C). Few genera of this phylum are known opportunistic pathogens^71^; however, studies on the association of members of this phylum longitudinally with age can give more insights into their mutualistic or pathogenic role. Further, we observed a negative correlation in the abundance of Bacteroides with age (Fig. 6B); this is in contrast to previous studies demonstrating the higher abundance of genus *Bacteroides* with increasing age^72,73^. With the increasing age, physiological changes occur in the oral cavity like thinning of oral mucosa, smooth and loosened stippling aspect, narrowing and alteration of the gingival epithelium, modification of epithelial-connective interface and decreasing of keratinization^74^. Here, *Granulicatella* and *Streptococcus* abundance decreased with age (Fig. 6D,E) while *Treponema* and *Fusobacterium* abundance increased with age (Fig. 6F,G). *Granulicatella* is the component of normal oral flora and *Streptococcus* is also normal flora and early colonizers of the oral microbial community. On the contrary, few members of the genera *Treponema* and *Fusobacterium* are opportunistic pathogens. These age-related changes could be associated with the physiological changes in the oral cavity with the increasing age.

This study expressly describes the age-related changes in the microbiome. However, analysis of hematological and biochemical parameters of blood may have further provided an opportunity to understand its association with the microbiome, the clear picture on age-related changes in the overall metabolism and health and disease status. Further studies with additional samples and multoimics approach can help strengthen these findings.

In conclusion, this study particularly highlights the precise and perceptible association of age with the microbiome. Our finding suggests that the age-related changes are very specific and bacterial phylum Proteobacteria needs to be investigated in detail to understand its specific physiological role in gut microbiome. Similarly, bacterial taxa, including *Treponema*, *Fusobacterium*, *Granulicatella* and *Streptococcus* the member of the human oral microbiome, can be explored for their importance in the oral microbiome. Also, the findings suggest that core taxa constitute more than 75% of the gut and oral microbiome, while only 67% of the skin microbiome, indicating a larger variability of the microbiome present on the skin. We present baseline data of the human microbiome from a healthy Indian sub-population, which could be used as a reference for further studies, including diabetes^75–77^ obesity and inflammatory diseases.

## Methods

### Ethical clearance declaration

The study was approved by the ethics committees of the National Centre for Cell Science (NCCS), Pune and King Edward’s Memorial Hospital Research Centre (KEMHRC), Pune. Written informed consent from the study subjects or their parents wherever applicable were taken, as per the guidelines of the institutional ethics committee and Indian Council of Medical Research (ICMR), India. We confirm that all the experiments were performed as per the approved guidelines.

### Recruitment of subjects

Subjects were recruited from the Vadu Health and Demographic Surveillance System (Vadu HDSS) area of the Vadu Rural Health Program, KEM Hospital Research Centre, Pune (VRHP, KEMHRC, Pune). The Vadu study population comprises of about 170,000 individuals that reside in 22 villages. The objective of Vadu HDSS is to create a longitudinal database of demographic information, including fertility, mortality, migration and marital status, of the Vadu area. Two villages, namely Dongargaon (Latitude: 18.7442326, Longitude: 73.4504317) and Shikrapur (Latitude: 18.687639, Longitude: 74.125671) were selected out of the total Vadu HDSS region.

The recruitment was done based on the following criteria.

1. Minimum three generations (I- age >50 years, II- age between 25 to 40 years and III- age between 3 to 15 years) with at least two members per generation must be living together in the same house structure.
2. Self-declared healthy individuals.
3. Families with individuals having a history of consumption of alcohol, tobacco and recent (last 3 months) use of antibiotics were excluded from the study.

Among the 30 families screened, six families comprising of 54 individuals fulfilled the required criteria and were included in the study.

### Metadata and sample collection

A Food Frequency Questionnaire (FFQ), along with 48 hrs dietary recall was administered before sample collection. Detailed information on the consumption of the food item and quantity for each meal of the day were recorded. With the help of nutritionists, this information then translated into the daily consumption of carbohydrates, proteins, lipids, fibers and calories. Additional metadata about the use of antibiotics and medicines, hygiene and sanitary practices, lifestyle, socioeconomic status, social habits, health and diseases (self-reported with or without medical records based on standard questionnaire) and other demographic characteristics were recorded. Separate health status questionnaires for adults and children were administered for the selection of healthy adults for the study (Tables S8 and S9).

Detailed information on routine dietary consumption of different food nutriments, their frequency and quantity were collected and recorded from the study participants. This information was recorded for three important meals, i.e., breakfast, lunch, and dinner. Also, the data on routine consumption of any additional specific food nutriment besides these three meals was recorded. The information on routine dietary consumption was then used for calculating the routine consumption of carbohydrates, proteins, lipids, fibers, and calories.

Gut, oral, and skin samples from the recruited subjects were collected in triplicates (with an interval of one week). Freshly voided, early morning fecal sample was collected in a sterile container. Early morning oral washing (before brushing or gargling) was collected using freshly prepared sterile 1X PBS (pH 7.4) in a sterile container. Skin samples from 11 different body sites per individual (belonging to three different regions, i.e., moist, oily and sebaceous region) were collected as described in Fig. S7. All samples were stored at −80 °C until further processing.

### Microbiome profiling

DNA extraction from fecal (representative of the gut), oral and skin samples was done using QIAamp stool DNA mini kit, QIAamp DNA mini kit, and QIAamp blood and tissue DNA extraction kit, respectively (Qiagen, USA). The DNA extraction was performed according to the manufacturer’s instruction with the inclusion of bead beating and freeze-thaw treatment at −80 °C and 90 °C for 10 minutes alternatively. Metagenomic sequencing of the V3-V4 region of the 16S rRNA gene was done using Illumina Miseq platform, paired-end (2 × 300 bp) sequencing, as described earlier^78^.

### Bioinformatics and statistical analysis

Assembly of paired-end reads for each sample was carried out using FLASH (Fast Length Adjustment of SHort reads). Low-quality sequences were removed during the assembly with low overlapping regions (less than 20 nucleotides)^79^. Microbial diversity analysis was done using standard QIIME (v1.8.0) pipeline^80^. Closed reference-based OTU picking approach was used to cluster reads into Operational Taxonomic Units (OTUs) at 97% sequence similarity using UCLUST algorithm^81^ and Greengenes database (13.8) and representative sequences from each OTU were selected for taxonomic assignment. Beta diversity and other statistical analysis was performed using Phyloseq^82^, corrplot^83^, vegan^84^ and Microbiome^85^ packages in R. Additional statistical analysis were performed using STAMP^86^ and GraphPad Prism (GraphPad Software, La Jolla California USA). A web-based tool InteractiVenn was used for the analysis of shared and unique bacterial genera^87^.

### Quantification of genus *Prevotella* in study population

Quantitation of genus *Prevotella* and total bacteria from fecal samples was carried out using qPCR as described previously^88^. Briefly, for quantifying 16S rRNA gene for total bacteria and *Prevotella*, 10 μl reactions in triplicate were set containing a suitable pair of primers^88^ (Table 2), 50 ng of Metagenomic DNA and SYBR green master mix (Applied Biosystems Inc. USA), using 7300 Real-time PCR system (Applied Biosystems Inc. USA). Following PCR conditions: initial denaturation at 95 °C for 10 min, followed by 40 cycles at 95 °C for 10 s, 60 °C for 1 min was used. Group-specific standard curves were generated from serial dilutions of a known concentration of respective PCR products. Additionally, melting curve analysis was performed at the end of qPCR cycles to check the amplification specificity. Average values of the triplicate were used for enumerations of tested gene copy numbers for each group using standard curves.

**Table 2:** Details for the qPCR primers and their amplicon size.

Sr. No	Bacterial taxa	Primers	Sequence (5′-3′)	Amplicon size (bp)
1	Total bacteria	341F	CCTACGGGAGGCAGCAG	177
		518R	ATTACCGCGGCTGCTGG	
2	Prevotella	PrevF	CACCAAGGCGACGATCA	283
		PrevR	GGATAACGCCYGGACCT	

## Supplementary information

Supplementary information.

## Data Availability

The sequence data is available at NCBI SRA submission with accession number SRP116277 (Bioproject ID: PRJNA399246) for gut microbiome, SRP135853 (Bioproject ID: PRJNA438584) for skin microbiome and SRP135913 (Bioproject ID: PRJNA438728) for oral microbiome.
