# Supplementary material for: Gut, oral and skin microbiome of Indian patrilineal families reveal perceptible association with age
Source: Sci Rep. 2020 Mar 30;10:5685. doi: 10.1038/s41598-020-62195-5 (PMC7105498; doi:10.1038/s41598-020-62195-5)
Supplement: Supplementary file 1 — Supplementary information. [file 41598_2020_62195_MOESM1_ESM.pdf]

Supplementary information

Gut, oral and skin microbiome of Indian patrilineal families reveal perceptible association with age

Diptaraj S. Chaudhari[1,3†], Dhiraj P. Dhotre[1†]*, Dhiraj M. Agarwal[2], Akshay H. Gaike[1,2], Devika Bhalerao[2], Parmeshwar Jadhav[2], Dattatray Mongad[1], Himangi Lubree[2], Vilas P. Sinkar[1], Ulhas K. Patil[3,6], Sundeep Salvi[4], Ashish Bavdekar[5], Sanjay K. Juvekar[2], Yogesh S. Shouche[1]*

[1]National Centre for Microbial Resource, National Centre for Cell Science, Central Tower, Sai Trinity Building Garware Circle, Sutarwadi, Pashan, Pune, India

[2]Vadu Rural Health Program, KEM Hospital Research Centre, Pune, India

[3]R. C. Patel ASC College, Shirpur, Dhule, India

[4]Chest Research Foundation, Marigold Premises, Survey no 15, Kalyaninagar, Pune, India

[5]KEM Hospital, Pediatrics Department, KEM Hospital, Rasta Peth, Pune, India

[6]Department of Microbiology, Government Institute of Science, Aurangabad, India

†Authors contributed equally

*** Correspondence:**

Yogesh S Shouche

Email: yogesh@nccs.res.in

Dhiraj P Dhotre

Email: dhiraj@nccs.res.in

Sequence of supplementary files

1. Fig. S1
2. Fig. S2
3. Fig. S3
4. Fig. S4
5. Fig. S5
6. Fig. S6
7. File S1
8. Table S1
9. Table S2
10. Table S3
11. Table S4
12. Table S5
13. Table S6
14. Table S7
15. Table S8
16. Table S9

Figure S1

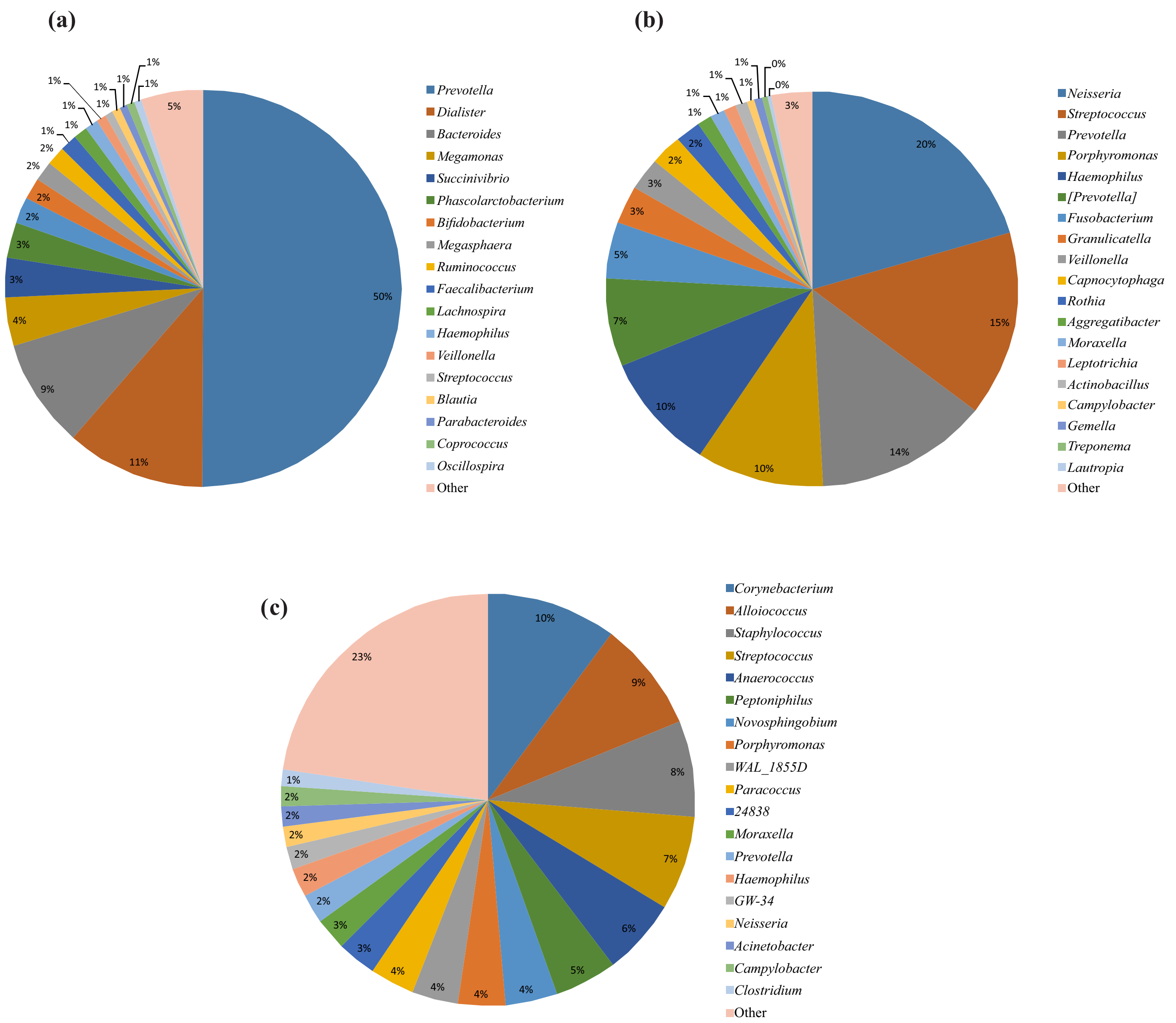

(a)
50%
11%
9%
4%
3%
5%
Prevotella
Dialister
Bacteroides
Megamonas
Succinivibrio
Phascolarctobacterium
Bifidobacterium
Megasphaera
Ruminococcus
Faecalibacterium
Lachnospira
Haemophilus
Veillonella
Streptococcus
Blautia
Parabacteroides
Coprococcus
Oscillospira
Other
(b)
20%
15%
14%
10%
10%
7%
5%
3%
3%
Neisseria
Streptococcus
Prevotella
Porphyromonas
Haemophilus
[Prevotella]
Fusobacterium
Granulicatella
Veillonella
Capnocytophaga
Rothia
Aggregatibacter
Moraxella
Leptotrichia
Actinobacillus
Campylobacter
Gemella
Treponema
Lautropia
Other
(c)
10%
9%
8%
7%
6%
5%
23%
Corynebacterium
Alloiococcus
Staphylococcus
Streptococcus
Anaerococcus
Peptoniphilus
Novosphingobium
Porphyromonas
WAL_1855D
Paracoccus
24838
Moraxella
Prevotella
Haemophilus
GW-34
Neisseria
Acinetobacter
Campylobacter
Clostridium
Other

Fig. S1: Pie charts showing the distribution of gut (a) oral (b) and (c) skin microbiome of endogamous agriculturist Indian (EAI) sub-population.

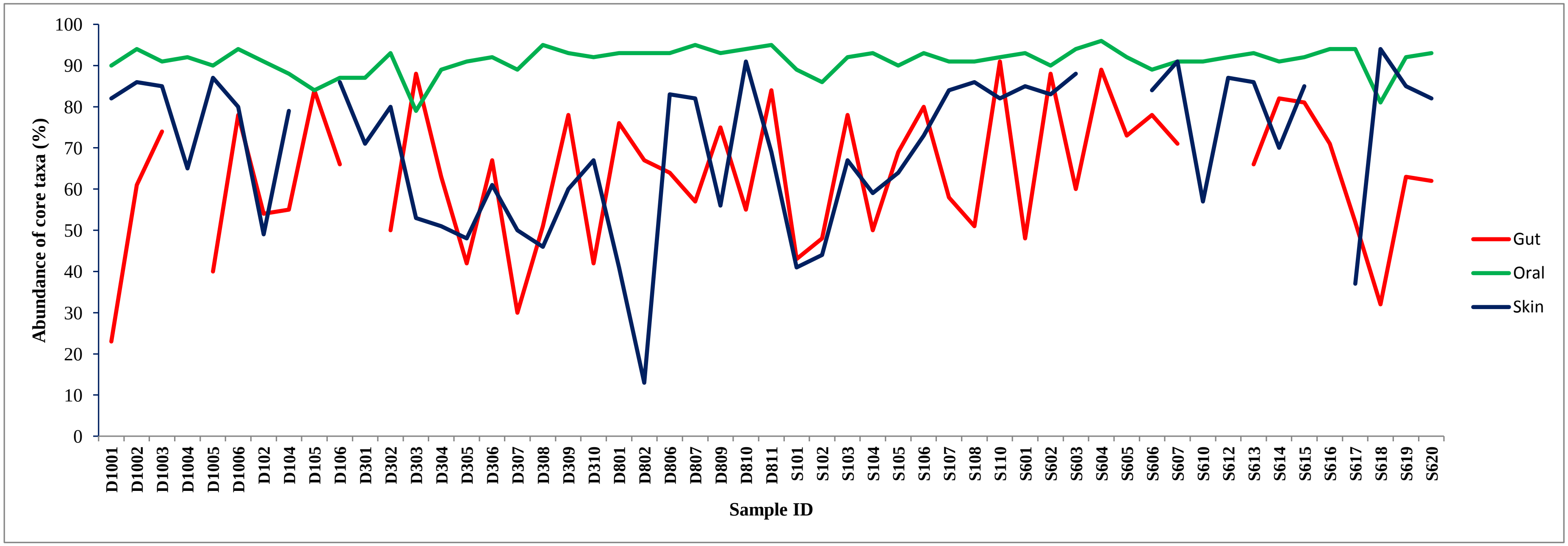

Abundance of core taxa (%)
100
90
80
70
60
50
40
30
20
10
0
D1001 D1002 D1003 D1004 D1005 D1006 D102 D104 D105 D106 D301 D302 D303 D304 D305 D306 D307 D308 D309 D310 D801 D802 D806 D807 D809 D810 D811 S101 S102 S103 S104 S105 S106 S107 S108 S110 S601 S602 S603 S604 S605 S606 S607 S610 S612 S613 S614 S615 S616 S617 S618 S619 S620
Sample ID
Gut
Oral
Skin

Fig. S2: Total proportion of microbiome represented by the core genera in the gut, oral and skin samples.

Figure S3

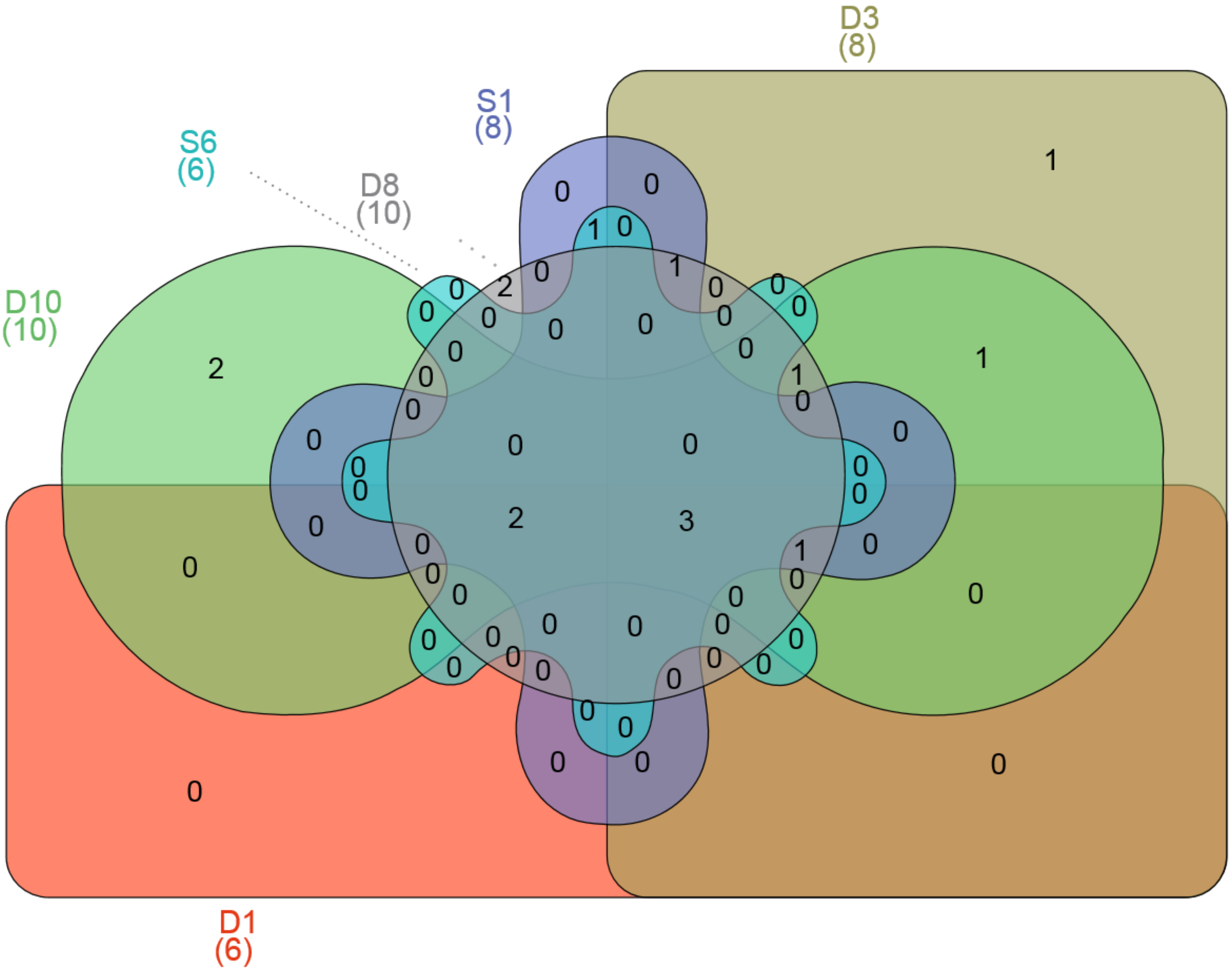

Fig. S3: Venn diagram representing the number of core genera detected in the gut microbiome across different families (D1, D3, D8, D10, S1 and S6) of the EAI population.

Figure S4

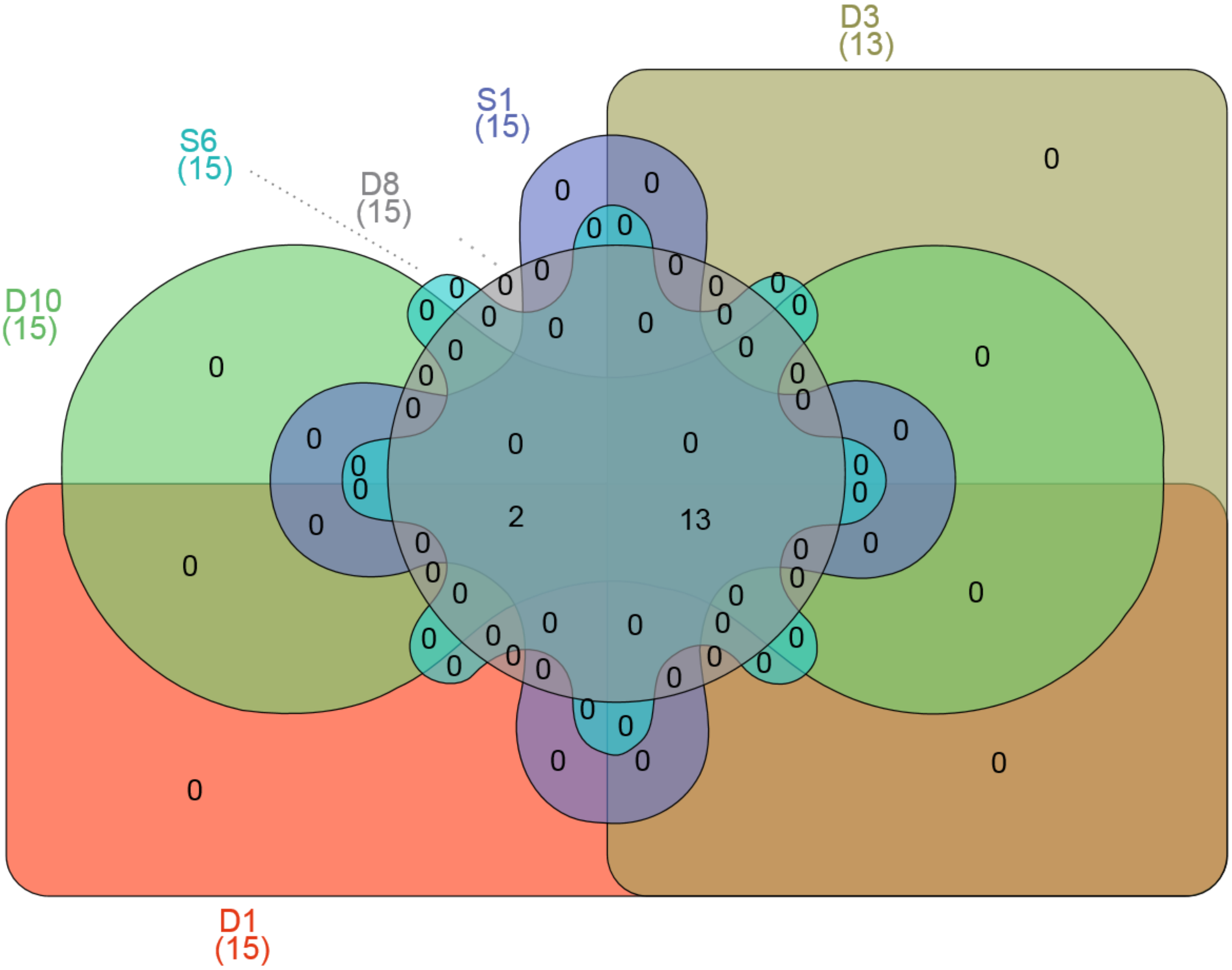

Fig. S3: Venn diagram representing the number of core genera detected in the oral microbiome across different families (D1, D3, D8, D10, S1 and S6) of the EAI population.

Figure S5

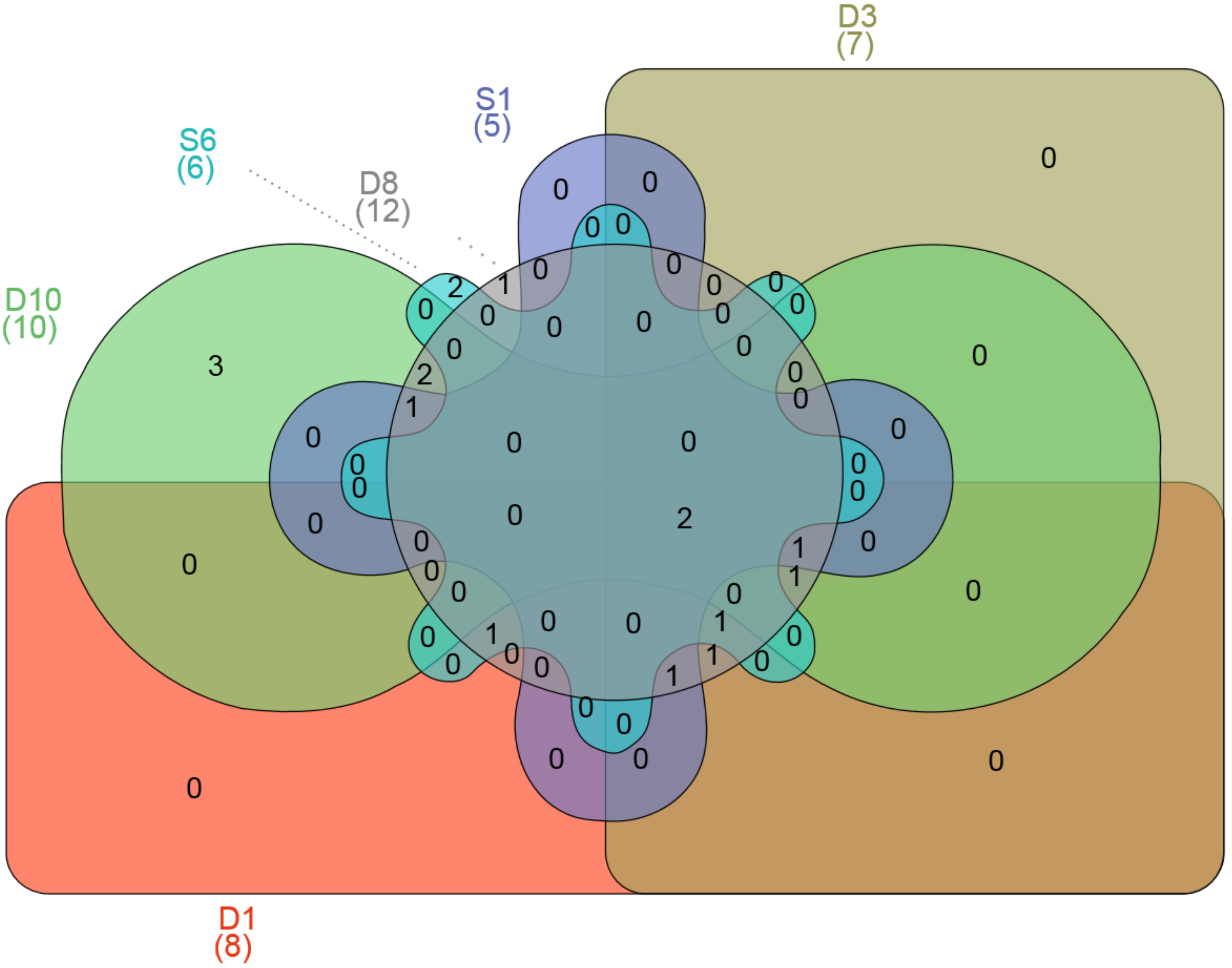

Fig. S3: Venn diagram representing the number of core genera detected in the skin microbiome across different families (D1, D3, D8, D10, S1 and S6) of the EAI population.

Figure S6

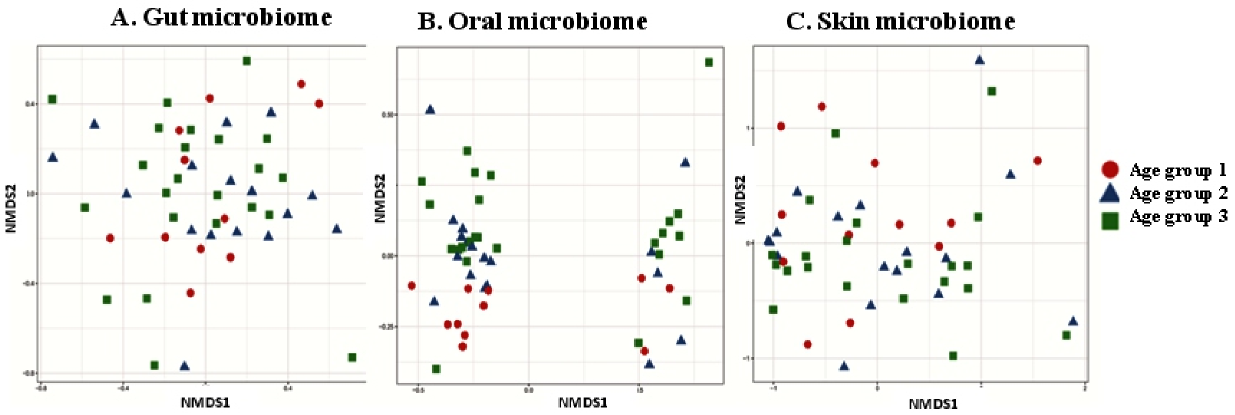

A. Gut microbiome
B. Oral microbiome
C. Skin microbiome
NMDS1
NMDS2
Age group 1
Age group 2
Age group 3

Fig. S6: Nonmetric Multidimensional Scaling (NMDS) ordination displaying microbiome communities across the three generations in the gut (A), oral (B) and skin (C) microbiome.

File S1: Number of reads (Raw, assembled and OTUs assigned reads) generated for the gut, oral and skin microbiome samples.

Sample ID	Number of raw reads			Number of assembled reads			Number of assigned sequences		
	**Stool**	**Skin**	**Oral**	**Stool**	**Skin**	**Oral**	**Stool**	**Skin**	**Oral**
**D1001**	85222	838877	273129	81318	642368	174599	74512	33759	66601
**D1002**	242000	1114624	415488	230028	823559	313673	196177	30731	211905
**D1003**	177543	846724	259749	168800	630186	177581	151251	130492	111265
**D1004**	20402	1301504	390717	19274	1079141	294759	17666	496624	173719
**D1005**	171130	988350	436848	160789	681705	202272	145667	441636	192756
**D1006**	263215	1422421	355424	250744	1053644	267825	224079	222360	147195
**D101**	241550	1507520	271128	231975	1231932	88554	212740	530844	15734
**D102**	344423	912277	386436	328884	689273	263056	302709	312747	157196
**D104**	282276	910947	260932	271317	641316	184397	229962	42618	111938
**D105**	289012	NA	497460	274494	NA	238674	236554	NA	219214
**D106**	259106	240897	489100	245941	122748	405968	223662	94264	290087
**D301**	334260	334566	308494	316871	216755	223471	277391	131601	101047
**D302**	341099	301205	342025	321124	214781	167487	270044	144896	157792
**D303**	133541	264044	382062	127082	172766	192094	104544	128389	178385
**D304**	220115	223940	258095	209618	96108	184922	152403	48080	104873
**D305**	174855	206714	332833	166469	121377	129687	136188	95009	120499
**D306**	112893	167665	517506	98413	89172	247090	76100	78743	235052
**D307**	224487	198448	487678	207971	92859	227140	173251	55643	214225
**D308**	183092	224770	247630	173980	103407	102774	159275	57245	97196
**D309**	177964	240195	263784	168064	103969	73222	141802	71164	68831
**D310**	148002	385127	503633	139337	251390	231044	122583	200253	219969
**D801**	188301	380675	514804	178898	247859	240205	144833	176812	226761
**D802**	178056	301748	248965	170606	200702	98487	141314	87022	91191
**D806**	150523	210506	427115	143269	116828	306581	115932	96713	181672
**D807**	142257	246198	262009	134357	116837	117761	118920	104850	111648
**D809**	230314	289951	264667	217163	147102	123039	171355	112054	116709
**D810**	219825	312907	268341	211599	253280	178108	195811	82755	74402
**D811**	142780	334739	320390	135548	185974	124315	104864	155555	116804
**S101**	206260	260555	283784	186710	116477	211450	129819	63845	121281
**S102**	146185	220567	422436	139268	78478	164416	121614	38203	102086
**S103**	217348	825205	370459	206397	673563	256842	157350	324636	184140
**S104**	99490	1080050	243694	95024	620456	136261	81824	315387	75187
**S105**	NA	1929796	243694	NA	1075923	181220	NA	396857	85064
**S106**	164883	1341746	299166	153617	1029779	211416	131992	351335	125035
**S107**	139551	646710	172347	130840	485921	74491	105970	50401	41581
**S108**	223597	683469	355289	213332	461338	133953	185055	117834	78269
**S110**	156998	684294	300429	149497	501258	119431	133560	248548	56769
**S601**	180199	1043725	231559	170007	710997	82112	122064	46477	43058
**S602**	220596	1294399	298481	212037	964509	251296	184745	627841	183310
**S603**	219655	808892	383040	207638	628902	333826	172554	370807	243632
**S604**	211020	NA	256502	198885	NA	174321	186274	NA	128025
**S605**	180050	1048	328205	169711	760	147617	155234	262	102828
**S606**	215603	1248686	256502	205674	938644	207052	180277	608786	126383
**S607**	148983	698308	419759	142649	558944	281266	100441	255981	236994
**S610**	11789	198705	255723	11137	128400	183081	10260	81974	95430
**S612**	NA	515354	289794	NA	354719	209282	NA	109073	92678

Sample ID	Number of raw reads			Number of assembled reads			Number of assigned sequences		
	**Stool**	**Skin**	**Oral**	**Stool**	**Skin**	**Oral**	**Stool**	**Skin**	**Oral**
**S613**	214633	333147	246693	203469	270657	155891	166136	101120	72718
**S614**	184295	2029325	363953	162105	727469	274272	136450	286194	194697
**S615**	189391	945646	387094	179712	724773	254223	159257	287644	211195
**S616**	201607	451859	542488	190841	243915	236303	151651	13745	224183
**S617**	197529	1677236	505983	186510	1355922	218850	150288	937261	211958
**S618**	267661	1127454	453323	254498	864711	295853	228777	415724	247330
**S619**	212554	1200894	245637	202980	963814	190024	145421	614844	112354
**S620**	224383	249020	270308	210026	154682	102925	130373	123537	57798

Table S1: Relative abundance of genus *Prevotella* in the gut microbiome of the EAI sub-population

Sample Id	Abundance (%)	Sample Id	Abundance (%)	Sample Id	Abundance (%)
St.S604	77	St.D102	49	St.S108	26
St.D811	73	St.S615	48	St.D302	26
St.D105	67	St.S103	47	St.S104	25
St.S110	67	St.D1002	46	St.D308	24
St.D809	64	St.D802	43	St.S101	23
St.D101	64	St.D1003	41	St.S601	22
St.D1006	62	St.S619	41	St.S607	22
St.S614	61	St.S613	38	St.S618	19
St.S106	60	St.D810	36	St.D304	13
St.S605	58	St.S105	36	St.D305	11
St.S606	55	St.D306	36	St.D1001	11
St.S602	55	St.D104	33	St.S107	8
St.D801	55	St.S617	33	St.D307	7
St.D106	54	St.D806	33	St.D310	6
St.S620	52	St.S102	29	St.D1005	2
St.D303	51	St.S603	28	St.D309	2
St.S616	49	St.D807	27		

Table S2: Relative abundance of genus *Prevotella* in the oral microbiome of the EAI sub-population.

Sample Id	Abundance (%)	Sample Id	Abundance (%)	Sample Id	Abundance (%)
or.S604	37	or.D310	12	or.D802	8
or.S619	37	or.D1004	11	or.D806	8
or.S603	35	or.D105	11	or.D1006	7
or.S620	34	or.S103	11	or.S606	7
or.S602	33	or.D306	10	or.S607	7
or.D1001	28	or.D807	10	or.D811	6
or.S617	28	or.S104	10	or.S610	6
or.D102	21	or.S105	10	or.D307	5
or.S601	21	or.S615	10	or.D809	5
or.D301	19	or.S106	9	or.S107	5
or.S613	17	or.S612	9	or.S618	5
or.S110	16	or.S614	9	or.D1003	4
or.S101	15	or.D1002	8	or.D303	4
or.S108	14	or.D1005	8	or.D104	3
or.S616	14	or.D106	8	or.D308	2
or.D302	13	or.D304	8	or.D810	2
or.S102	13	or.D305	8	or.D309	0
or.S605	13	or.D801	8		

Table S3: Core bacterial genera present in the gut microbiome of overall EAI study population and across the members of six different families.

Sr. No	Core genera	EAI Study population	Families					
			D1	D3	D8	D10	S1	S6
1	*Prevotella*	+	+	+	+	+	+	+
2	*Ruminococcus*	+	+	+	+	+	+	+
3	*Faecalibacterium*	+	+	+	+	+	+	+
4	*Bacteroides*	+	+	+	+	+	+	-
5	*Lachnospira*	+	+	-	+	+	+	+
6	*Coprococcus*	+	+	-	+	+	-	+
7	*Blautia*	-	-	+	+	+	-	-
8	*Bifidobacterium*	-	-	+	-	+	-	-
9	*Oscillospira*	-	-	+	+	-	-	-
10	*Dialister*	-	-	-	-	-	+	+
11	*Parabacteroides*	-	-	+	-	-	-	-
12	*Haemophilus*	-	-	-	+	-	-	-
13	*Roseburia*	-	-	-	+	-	-	-
14	*Streptococcus*	-	-	-	-	+	-	-
15	*Dorea*	-	-	-	-	+	-	-

$^{+}$Presence of bacterial taxa; $^{-}$Absence of bacterial taxa

Table S4: Core bacterial genera present in the oral microbiome of overall EAI study population and across the members of six different families.

Sr. No	Core genera	EAI Study population	Families					
			D1	D3	D8	D10	S1	S6
1	*Neisseria*	+	+	+	+	+	+	+
2	*Streptococcus*	+	+	+	+	+	+	+
3	*Prevotella*	+	+	+	+	+	+	+
4	*Porphyromonas*	+	+	+	+	+	+	+
5	*Haemophilus*	+	+	+	+	+	+	+
6	*Fusobacterium*	+	+	+	+	+	+	+
7	*Granulicatella*	+	+	+	+	+	+	-
8	*Veillonella*	+	+	+	+	+	+	-
9	*Capnocytophaga*	+	+	+	+	+	+	-
10	*Rothia*	+	+	+	+	+	+	-
11	*Aggregatibacter*	+	+	+	+	+	+	-
12	*Gemella*	+	+	+	+	+	+	-
13	*Lautropia*	-	+	+	+	+	+	-
14	*Leptotrichia*	+	+	-	+	+	+	-
15	*Campylobacter*	+	+	-	+	+	+	-

$^{+}$Presence of bacterial taxa; $^{-}$Absence of bacterial taxa

Table S5: Core bacterial genera present in the skin microbiome of overall EAI study population and across the members of six different families.

Sr. No	Core genera	EAI Study population	Families D1	D3	D8	D10	S1	S6
1	*Corynebacterium*	+	+	+	+	+	+	+
2	*Streptococcus*	+	+	+	+	+	+	+
3	*Staphylococcus*	+	+	+	+	+	+	-
4	*Porphyromonas*	+	+	+	+	-	-	+
5	*Paracoccus*	+	+	+	+	-	+	-
6	*Acinetobacter*	+	+	+	+	+	-	-
7	*Anaerococcus*	+	+	+	+	-	-	-
8	*Prevotella*	+	+	-	+	-	-	+
9	*Pseudomonas*	-	-	-	+	+	+	-
10	*Alloiococcus*	-	-	-	+	+	-	-
11	*Bacillus*	-	-	-	+	+	-	-
12	*Novosphingobium*	-	-	-	+	-	-	-
13	*Enhydrobacter*	-	-	-	-	+	-	-
14	*Salinicoccus*	-	-	-	-	+	-	-
15	*Butyrivibrio*	-	-	-	-	+	-	-
16	*Haemophilus*	-	-	-	-	-	-	+
17	*Gemella*	-	-	-	-	-	-	+

$^{+}$Presence of bacterial taxa; $^{-}$Absence of bacterial taxa

Table S6: Dietary details of the study population characterized by daily intake of the dietary carbohydrates, fats, proteins, fibers and energy in the three generation members of the EAI population.

Family	Generation	Number of participants	Average age (years)	Daily intake of the food components Carbohydrates (gms)	Energy (Kcal)	Fat (gms)	Fiber (gms)	Proteins (gms)
**D1**	Generation one	02	54(±4.9)	93.6±7.7	606.6±40.4	12.7±0.8	14.4±2.5	18.9±0.6
	Generation two	01	29	664.9±0	3157.2±0	52.9±0	56.4±0	108.5±0
	Generation three	02	13(±1.4)	468.6±0	2239.2±0	37.2±0	42.4±0	73.5±0
	**Total**	**05**		**357.8±254.2**	**1769.8±1126.2**	**30.5±17.5**	**34±18.8**	**58.6±39**
**D3**	Generation one	02	55(±4.24)	94.2±5.8	633.5±2	16±0.8	21.2±2.1	16.9±2
	Generation two	02	29(±3.95)	254.6±279.7	1341±1267.1	27±19.6	28.9±19.2	42.1±44.1
	Generation three	02	8(±3.4)	286.6±87.1	1432.2±393.7	27.3±5.6	26.3±10.8	47.4±12.6
	**Total**	**06**		**235.3±185.4**	**1235.9±830.3**	**24.9±12.7**	**26.3±13.1**	**39.2±29.1**
**D8**	Generation one	02	53(±4.24)	153.5±61.6	873±306.2	17±5.7	24.2±6.4	24.4±14.8
	Generation two	04	27	56.8±0	336.8±0	8±0	15.6±0	6.2±0
	Generation three	04	10(±2.9)	295.5±23.7	1522.2±122.6	26.2±2.2	28.6±3.7	52.9±3.5
	**Total**	**10**		**220.8±103.1**	**1167.4±500.9**	**21±7.7**	**25.5±6.1**	**38.1±20.5**
**D10**	Generation one	02	54(±5.6)	76.1±0.7	438.2±3.7	10±0.1	13.9±2.5	7.7±0.1
	Generation two	01	27.5(±3.5)	230.3±66.3	1174.5±227.1	22.7±1.8	28.5±5.3	37.1±0.8
	Generation three	04	5.5(±0.7)	181.8±35.5	1006.5±158.1	18.9±2.5	16±2.9	40.9±5.3
	**Total**	**07**		**162.7±78.1**	**873±366.6**	**17.2±6**	**19.5±7.7**	**28.6±16.4**
**S1**	Generation one	02	64(±5.6)	347.3±170.1	1634.1±822.9	27.1±13.3	28.2±15.2	51.5±26.4
	Generation two	03	38(±3.41)	515±89.2	2442.5±418.4	41.2±5.9	44.6±5.3	80±14.9
	Generation three	04	11.5(±2.38)	383.4±88.4	1864.8±398.9	30.3±5.5	31.9±7.3	60.9±13.2
	**Total**	**09**		**419.2±117.9**	**2006.1±551.4**	**33.3±8.9**	**35.3±10.3**	**65.2±18.6**
**S6**	Generation one	02	65(±7.07)	202±136.2	1052.6±564.8	18.8±7.2	23.1±8.8	27.2±18.1
	Generation two	07	34.5(±3.04)	458.7±122.7	2232.8±591.3	38.7±11.3	42.2±10.9	70.7±22
	Generation three	08	10.8(3.4±)	376.2±123.8	1859.2±569.7	32.5±9.5	34.3±10	60.7±19.9
	**Total**	**17**		**389.6±141.7**	**1918.2±657.1**	**33.4±11.4**	**36.3±11.5**	**60.8±23.7**

*gms=grams

Table S7a: Mean relative abundance of prevalent bacterial taxa detected across the three age groups in the gut microbiome.

Sr. No.	Bacterial taxa	Mean relative abundance of bacterial taxa (gut microbiome)		
		Age group 1	Age group 2	Age group 3
1	*Prevotella*	38.46	36.68	39.34
2	*Dialister*	8.53	9.19	8.40
3	*Bacteroides*	1.94	6.34	9.41
4	*Megamonas*	2.96	4.64	1.99
5	*Succinivibrio*	6.33	1.98	0.98
6	*Phascolarctobacterium*	1.18	2.88	2.21
7	*Megasphaera*	1.65	1.69	0.69
8	*Ruminococcus*	1.44	0.95	1.21
9	*Bifidobacterium*	0.41	1.33	1.69
10	*Faecalibacterium*	0.80	1.31	1.09

Table S7b: Mean relative abundance of prevalent bacterial taxa detected across the three age groups in the oral microbiome.

Sr. No.	Bacterial taxa	Mean relative abundance of bacterial taxa (oral microbiome)		
		Age group 1	Age group 2	Age group 3
1	*Neisseria*	18.22	17.72	19.68
2	*Prevotella*	16.95	11.91	10.86
3	*Streptococcus*	8.62	13.52	16.46
4	*Porphyromonas*	7.43	8.88	10.09
5	*Haemophilus*	6.42	10.32	8.71
6	*Fusobacterium*	6.23	4.76	3.02
7	*Granulicatella*	1.86	2.56	3.38
8	*Veillonella*	3.30	2.22	2.19
9	*Capnocytophaga*	3.05	2.58	1.72
10	*Rothia*	2.00	2.00	1.70

Table S7c: Mean relative abundance of prevalent bacterial taxa detected across the three age groups in the skin microbiome.

Sr. No.	Bacterial taxa	Mean relative abundance of bacterial taxa (skin microbiome)		
		Age group 1	Age group 2	Age group 3
1	*Corynebacterium*	10.95	10.02	5.53
2	*Alloiococcus*	10.74	3.12	8.30
3	*Staphylococcus*	5.15	4.71	7.96
4	*Anaerococcus*	5.14	8.18	2.17
5	*Peptoniphilus*	4.66	3.58	4.02
6	*Haemophilus*	2.49	0.90	2.31
7	*Porphyromonas*	2.09	4.82	2.13
8	*Clostridium*	2.04	0.68	0.92
9	*Novosphingobium*	1.91	3.02	4.37
10	*Streptococcus*	1.38	3.90	10.47
11	*Acinetobacter*	1.37	1.24	1.30
12	*Prevotella*	1.35	1.34	2.62
13	*Campylobacter*	1.34	1.89	0.67

Table S8: Health Status Questionnaire (Adult)

Study ID: □□□□□□ HDSS ID:□□□□□□□□□□□□		
Date: □□/□□/□□□□		
Name of Study participant: ______________________________		
Q 1	Age	□□ Years
Q 2	Sex	01.Male<br>02.Female
Q3	Complaints if any	……………………………………<br>……………………………………<br>……………………………………<br>……………………………………<br>…………………
Q 4	H/O present complaints	……………………………………<br>……………………………………<br>……………………………………<br>……………………………………<br>…………………
Q 5	Past History of illness	……………………………………<br>……………………………………<br>……………………………
Q 6	History of medication/surgery	……………………………………<br>……………………………………<br>……………………………
Q 7	Family History of Disease if any:	……………………………………<br>……………………………………<br>……………………………
**If no obvious complain then ask further,**		
**Personal History**		
Q 8	Have you ever had difficulty in breathing?	01.Yes<br>02. No
Q 9	Do you have cough/ fever for more than 21 days?	01.Yes<br>02. No
Q 10	Have you ever had any kind of chest pain?	01.Yes<br>02. No
Q 11	Have you ever observed swelling on feet or face especially evening or in morning since last month?	01.Yes<br>02. No
Q 12	Have you ever felt sudden black out?	01.Yes<br>02. No
Q 13	Have you ever felt vertigo since one month?	01.Yes<br>02. No
Q 14	Do you think your thirst has increased these days?	01.Yes<br>02. No
Q 15	Do you feel that your appetite has become very low, very high or frequent since last month?	01.Yes<br>02. No
Q 16	Do you feel numbness or loss of sensation in any part of body?	01.Yes<br>02. No

Q 17	Have you ever had profuse sweating?	01.Yes<br>02. No
Q 18	If yes, specify if any other symptoms present at that time.	....
Q 19	Have you ever had severe or regular headache?	01.Yes<br>02. No
Q 20	Have you ever had an episode of convulsion?	01.Yes<br>02. No
Q 21	Is there any abnormal growth/mass in any part of your body?	01.Yes<br>02. No
Q 22	Do you feel sensation of vomiting before or after meal?	01.Yes<br>02. No
Q 23	Have you ever had unusual constipation or frequent loose stool since one month?	01.Yes<br>02. No
Q 24	Have you had acute /chronic/frequent abdominal pain?	01.Yes<br>02. No
Q25	Do you frequently feel a burning sensation in the stomach?	01.Yes<br>02. No
Q 26	Do you have pimples/ watery lesions/warts/wounds/white patches/rashes on your face, chest or arms?	01.Yes<br>02. No
Q 27	Do you frequently have excessive skin itching?	01.Yes<br>02. No
Q 28	Do you have frequent toothache or dental caries?	01.Yes<br>02. No
Q 29	Do you have any ulcer/lesion in the oral cavity?	01.Yes<br>02. No
Q 30	Have you experienced unusually frequent/ very low/ burning sensation<br>/ pain in urination recently/ in past one month?	01.Yes<br>02. No
Q 31	Do you feel that you sleep has become very less/ very high/ disturbed recently/ in last one month?	01.Yes<br>02. No
Q 32	Have you had bleeding from any part of your body (without injury)<br>in any form?	01.Yes<br>02. No
Q 33	Have you been absent from your work because of weakness?	01.Yes<br>02. No
Q34	Have you ever had any joint pain while trying to climb or at the time of mild exercise?	01.Yes<br>02. No
**For the female Study participants**		
Q 34	What was your last menstruation date?	□□/□□/□□□□
Q 35	Do you have unusually heavy/ scanty menstruation bleeding?	01.Yes<br>02. No
Q 36	Do you have irregular or painful menstruation?	01.Yes<br>02. No
Q 38	Do you have any abnormal vaginal discharge?	01.Yes<br>02. No
Name of the Interviewer:<br><br>Signature:		

Table S9: Health Status Questionnaire (Children)

Study ID: □□□□□□ HDSS ID:□□□□□□□□□□□□□		
Date: □□/□□/□□□□		
Name of Study participant: ____		
Q 1	Age	□□ Years
Q 2	Sex	01.Male<br>02.Female
Q3	Complaints if any	....<br>..<br>..<br>..<br>...
Q 4	Past H/o Illness / convulsions	....<br>..<br>...
Q 5	History of surgery	....<br>..<br>...
Q6	Any medication at present	
Q7	Vaccination	OPV- Yes/No
		BCG- Yes/No
		DPT- Yes/No
		HEP-B- Yes/No
		Measles- Yes/No
		Other specify..
Q 8	Family History of Disease if any:	....<br>..<br>.............................
**If no obvious complain then ask further,**		
**Personal History**		
Q 9	Does the child have headaches frequently?	01.Yes<br>02. No
Q 10	Does the child complain of breathing difficulty?	01.Yes<br>02. No
Q 11	Does the child snore/breathe through his mouth?	01.Yes<br>02. No
Q 12	Does the child have difficulty in swallowing/ complain of throat pain?	01.Yes<br>02. No
Q 13	Does the child have watery lesions/warts/wounds/white patches/rashes on the face, chest or arms?	01.Yes<br>02. No
Q 14	Does the child frequently have excessive skin itching?	01.Yes<br>02. No
Q 15	Does the child have any ulcer/lesion in the oral cavity?	01.Yes<br>02. No
Q 16	Has the Child’s appetite become very low/ very high	01.Yes

	since last month?	02. No
Q 17	Does the child complain of vomiting before or after meal?	01.Yes<br>02. No
Q 18	Has the child complained of acute or frequent abdominal pain since last month?	01.Yes<br>02. No
Q 19	Does the child have a habit of eating chalks or mud or any non-eatable substance?	01.Yes<br>02. No
Q 2O	Does the child have unusual constipation or frequent loose stool since last month?	01.Yes<br>02. No
Q 21	Have you observed passage of worms in stool of the child?	01.Yes<br>02. No
Q 22	Does the child have a habit of scratching the anus at night?	01.Yes<br>02. No
Q 23	Does the child complain of pain or burning during micturition?	01.Yes<br>02. No
Q 24	Has the child's sleep become very less/ very high/ disturbed recently/ in last one month?	01.Yes<br>02. No
Q 25	Does the child have Swelling on face/feet?	01.Yes<br>02. No
Q 26	Does the child complain of Chest pain?	01.Yes<br>02. No
Q 27	Does the child have fever/cough for more than 3 weeks?	01.Yes<br>02. No
Q 28	Does the child bleed from any part of the body (without injury) in any form?	01.Yes<br>02. No
Q 29	Did the child suddenly get unconscious/or have a blackout/Anytime?	01.Yes<br>02. No
Name of the Interviewer:<br><br>Signature:		
